# The Velvet Proteins VosA and VelB Play Different Roles in Conidiation, Trap Formation, and Pathogenicity in the Nematode-Trapping Fungus *Arthrobotrys oligospora*

**DOI:** 10.3389/fmicb.2019.01917

**Published:** 2019-08-20

**Authors:** Guosheng Zhang, Yaqing Zheng, Yuxin Ma, Le Yang, Meihua Xie, Duanxu Zhou, Xuemei Niu, Ke-Qin Zhang, Jinkui Yang

**Affiliations:** ^1^State Key Laboratory for Conservation and Utilization of Bio-Resources in Yunnan, Yunnan University, Kunming, China; ^2^School of Life Sciences, Yunnan University, Kunming, China; ^3^Key Laboratory for Microbial Resources of the Ministry of Education, Yunnan University, Kunming, China

**Keywords:** *Arthrobotrys oligospora*, velvet proteins, mutants, conidiation, trap formation, pathogenicity

## Abstract

The velvet family proteins VosA and VelB are involved in growth regulation and differentiation in the model fungus *Aspergillus nidulans* and other filamentous fungi. In this study, the orthologs of VosA and VelB, AoVosA, and AoVelB, respectively, were characterized in the nematode-trapping fungus *Arthrobotrys oligospora*, which captures nematodes by producing trapping devices (traps). Deletion of the *AovelB* gene resulted in growth defects in different media, and the aerial hyphae from the Δ*AovelB* mutant lines were fewer in number and their colonies were less dense than those from the wild-type (WT) strain. The Δ*AovelB* mutants each displayed serious sporulation defects, and the transcripts of several sporulation-related genes (e.g., *abaA*, *flbC*, *rodA*, and *vosA*) were significantly down-regulated compared to those from the WT strain. Furthermore, the Δ*AovelB* mutant strains became more sensitive to chemical reagents, including sodium dodecyl sulfate and H_2_O_2_. Importantly, the Δ*AovelB* mutants were unable to produce nematode-capturing traps. Similarly, extracellular proteolytic activity was also lower in the Δ*AovelB* mutants than in the WT strain. In contrast, the Δ*AovosA* mutants displayed no obvious differences from the WT strain in these phenotypic traits, whereas conidial germination was lower in the Δ*AovosA* mutants, which became more sensitive to heat shock stress. Our results demonstrate that the velvet protein AoVelB is essential for conidiation, trap formation, and pathogenicity in *A. oligospora*, while AoVosA plays a role in the regulation of conidial germination and heat shock stress.

## Introduction

Many filamentous fungi naturally produce asexual conidia, and these constitute the main reproductive propagule and infectious particles in these organisms. Conidia formation is a complicated biological process that requires the coordination and cooperation of many genes (Adams et al., 1998; Park and Yu, 2012). Multiple regulatory genes and pathways for conidia production in filamentous fungi have been reported, and many studies have been conducted using *Aspergillus* as the model fungal species (Park and Yu, 2012, 2016; Chang et al., 2013). The results from previous studies have indicated that conidiophore production is genetically regulated and controlled by multiple activators and repressors in filamentous fungi (Mirabito et al., 1989; Krijgsheld et al., 2013). These regulators govern the coordinated expression of distinct gene sets required for the progression of each stage (Etxebeste et al., 2010; Park and Yu, 2012). In the model fungi *Aspergillus nidulans* and the pathogenic fungus *A. fumigatus*, these regulators are divided into three categories: upstream regulators, central regulators, and feedback regulators (Park and Yu, 2012, 2016). The central regulatory pathway contains three key elements, *brlA*, *abaA*, and *wetA*, which coordinate conidiation-specific gene expression, whereas the upstream developmental activators *fluG*, *flbA*, *flbB*, *flbC*, *flbD*, and *flbE* initiate the sporulation process. Velvet family proteins VelB and VosA were identified as the feedback regulators for conidiation (Park and Yu, 2012, 2016).

The velvet regulators VeA, VelB, VelC, and VosA constitute a class of fungi-specific DNA-binding, velvet domain-containing transcription factors (Ahmed et al., 2013; Beyhan et al., 2013). Velvet family proteins share a homologous region of approximately 150 amino acids and were first discovered in *A. nidulans* (Ni and Yu, 2007; Park and Yu, 2016). In *Aspergillus* species, the velvet proteins coordinate fungal growth, development, pigmentation, and primary/secondary metabolism (Ni and Yu, 2007; Park et al., 2012a, b, 2014). Velvet proteins, such as VelB/VeA/LaeA, VelB/VosA, and VelB/VelB, can form multimeric velvet complexes that regulate different developmental processes (Bayram et al., 2008; Sarikaya Bayram et al., 2010). Among these complexes the VosA–VelB complex functions as a key functional unit controlling spore maturation, trehalose biosynthesis, and conidial germination (Sarikaya Bayram et al., 2010; Park et al., 2012b). VelB, a primary protein in the composition of velvet complexes, plays a positive role in regulating conidiation in *A. nidulans* and also plays an important role in its secondary metabolism (Bayram et al., 2008; Sarikaya Bayram et al., 2010). Recent studies have shown that VelB predominantly interacts with VosA in asexual spores and plays an interdependent role in conidial maturation and germination (Park et al., 2012b). These findings suggest that VosA and VelB play crucial roles in the production of asexual spores and conidial maturation.

Nematode-trapping fungi (NTF) are a special group of filamentous fungi by virtue of their innate abilities to capture and digest nematodes by producing traps (trapping devices). Sporulation plays an important role in the growth and reproduction of NTF. *Arthrobotrys oligospora* is a typical nematode-trapping fungal species that can complete its reproduction asexually by producing abundant conidia (Niu and Zhang, 2011). At present, little is known about the genes involved in conidiation or the related regulatory genes for spore production in *A. oligospora* and other NTF. In 2011, the genome of the fungus *A. oligospora* was sequenced (Yang et al., 2011), providing a good opportunity to elucidate the mechanism involved in conidiation in this fungus. Compared with the *A. nidulans* fungus, many homologous sporulation-related regulators, such as upstream regulators (FluG, FlbC, and FlbD) and feedback regulators (VosA and VelB), were found in *A. oligospora*. In the present study, we characterized the functions of the homologous VosA (AoVosA) and VelB (AoVelB) proteins in *A. oligospora* and investigated their roles in the regulation of several important phenotypic traits, including conidiation and cell wall synthesis, using quantitative reverse-transcription PCR (RT-PCR). The results show that AoVelB plays an important role in the growth, conidiation, trap formation, and pathogenicity in *A. oligospora*.

## Materials and Methods

### Fungal Strains and Culture Conditions

*Arthrobotrys oligospora* Fres. (ATCC 24927) and its derived fungal mutants were maintained on potato dextrose agar (PDA) medium at 28°C for standard culturing. Liquid TG (1% tryptone and 1% glucose) medium was used to collect the fungal mycelia. Plasmid pRS426 (a gift from Dr. K. A. Borkovich, University of California, Riverside, CA, United States) was maintained in the *Escherichia coli* strain DH5α (TaKaRa Bio, Shiga, Japan) and used to construct recombinational plasmids. The PDAS (PDA supplemented with 10 g/L molasses and 0.4 M saccharose) medium was used for protoplast regeneration. TG, TYGA, and CMY media were prepared as previously described (Yang et al., 2018) and were used to analyze the phenotypic traits of *A. oligospora* and its corresponding mutants. The nematode *Caenorhabditis elegans* was maintained on oatmeal water medium at 26°C.

### Sequence and Phylogenetic Analyses of AoVosA and AoVelB From *A. oligospora*

The VosA and VelB velvet proteins from *A. nidulans* were used to search for their homologous sequences in the *A. oligospora* genome. Their orthologs, AoVosA (AOL_s00054g700) and AoVelB (AOL_s00054g811), were downloaded from GenBank^1^. The AoVosA and AoVelB homologs from other filamentous fungi identified in GenBank were downloaded and used to construct neighbor-joining trees for VosA and VelB, respectively, using the Mega 7 software package (Kumar et al., 2016).

### Deletion of *AovosA* and *AovelB* Genes

The cetyltrimethylammonium bromide (CTAB) method was used to isolate genomic DNA from fungal *A. oligospora.* The replacement fragments from the *AovosA* and *AovelB* genes were constructed using a modified yeast cloning procedure (Colot et al., 2006). The 5′ and 3′ flanking sequences of the target genes and the *hph* cassette (Staben et al., 1989) were cloned from *A. oligospora* and pCSN44 with paired primers (Supplementary Table S1), respectively. These DNA fragments and the pRS426 plasmid backbone (digested with *Eco*RI and *Xho*I) were simultaneously transformed into *Saccharomyces cerevisiae* strain FY834 by electroporation (Winston et al., 1995). The constructed vectors (pRS426-AoVosA-hph and pRS426-AoVelB-hph) were maintained in *E. coli* DH5a (TaKaRa Bio) through transformation. Finally, the combined plasmids pRS426-AoVosA-hph and pRS426-AoVelB-hph were individually transformed into the *A. oligospora* protoplast as previously described (Yang et al., 2018; Zhen et al., 2018, 2019).

The transformants were screened for the presence of the *hph* resistance gene using hygromycin (200 μg/mL) (Tunlid et al., 1999). The putative transformants were confirmed as authentic by PCR amplification using specific primers (Yf and Yr) and Southern blot analyses with specific probes (Supplementary Table S1). Southern blotting was performed using the North2South Chemiluminescent Hybridization and Detection kit (Pierce, Rockford, IL, United States) according to the manufacturer’s instructions.

### Comparison of Mycelial Growth, Conidiation, and Morphology

After incubating the wild-type (WT) strain, Δ*AovosA* and Δ*AovelB* mutants on PDA plates at 28°C for 6 days, 7 mm diameter hyphal discs were punched from the edges of plate colonies to compare their growth characteristics in different media, including PDA, TYGA, and TG. After 5–8 days of incubation, the diameter and hyphal morphology of each colony were measured and observed. The WT and mutant strains that were centrally attached to the CMY plates were also cultivated at 28°C for 15 days. The number of conidia and their morphologies were next determined and observed as previously described (Zhen et al., 2018). The mycelia from the WT and mutants were treated according to the methods described previously (Kim et al., 2004). After critical-point drying using a model CPD-030 device (Bal-Tec AG, Balzers, Liechtenstein), the samples were coated with gold using a model SCD 005 sputter coater (Bal-Tec) and observed by scanning electron microscopy (Quanta-200; FEI, Hillsboro, OR, United States).

### Stress Tolerance Analysis

The responses of the WT and mutants to different chemical stressors, including osmotic, oxidative, or cell wall-perturbing stressors, were determined by incubating them on TG supplemented with different concentrations of NaCl (0.1, 0.2, and 0.3 M) and sorbitol (0.25, 0.50, and 0.75 M) for osmotic stress, hydrogen peroxide (H_2_O_2_; 5, 10, and 15 mM) and menadione (0.01, 0.03, and 0.05 mM) for oxidative stress, or sodium dodecyl sulfate (SDS; 0.01, 0.02, and 0.03%) and Congo red (0.05, 0.07, and 0.09 mg/mL) for cell wall perturbation (Yang et al., 2018; Zhen et al., 2018) at 28°C for 6 days. The diameter and hyphal morphology of each colony were measured and observed.

### Heat Shock Pressure Tolerance Test

To determine the recuperation of the WT strain and Δ*AovosA* mutants following heat shock, their growth rates were compared after incubation on PDA plates for 2 days at 28°C, and 8 h at 28, 34, 38, or 40°C followed by incubation at 28°C for 6 days, after which the colony diameter was determined (Zhen et al., 2019). For spore germination analysis under heat shock stress, 150 μL aliquots of conidial suspension (100 conidia) collected from the WT strain and Δ*AovosA* mutants was incubated in liquid MM medium at 28, 34, or 38°C for 4 and 8 h to assay conidial germination rates (Xie et al., 2012). Additionally, conidial suspension (100 conidia) collected from the WT strain and Δ*AovosA* mutants was incubated for 2 h at 28, 34, 38, or 42°C, then the spores were inoculated on TYGA plates and incubated at 28°C for 48 h. The surviving colonies were counted (Ni and Yu, 2007).

### Extracellular Protease Activity Analysis

WT and mutant strains were incubated on LMZ broth at 28°C for 7 days and the proteolytic activity of the fermentation liquids was assessed on casein plates as previously described (Zhao et al., 2004). Quantitative analysis of protease activity was determined by a caseinolytic method described by Wang et al. (2006). One unit (U) of protease activity was defined as the amount of enzyme that hydrolyzed the substrate and produced 1 μg of tyrosine in 1 min under the assay conditions.

### Trap Formation and Pathogenicity of *A. oligospora* Against Nematodes

WT and mutant strains were incubated on WA (water agar) plates at 28°C for 3–4 days. Approximately 300 nematodes (*C. elegans*) were added to the middle of each plate to induce trap formation in the fungi. The number of traps and captured nematodes were counted using a light microscope at specific time intervals (Yang et al., 2018).

### Quantitative Reverse-Transcription PCR (RT-PCR) Analysis

The WT strain and the Δ*AovelB* and Δ*AovosA* mutants were incubated in PD broth at 28°C for 3, 5, and 7 days. Hyphae were collected and stored at −80°C for subsequent RNA extraction. Total RNA from each sample was extracted using an RNA Extraction Kit (AP-MN-MS-RNA, Axygen, Jiangsu, China). The RNAs from the different samples were reverse transcribed into complementary DNAs (cDNAs) using the FastQuant RT Kit (TaKaRa Bio). The cDNAs from all the samples were used as templates to determine the transcriptional levels of the candidate genes (e.g., sporulation and protease-related genes) via RT-PCR with specific paired primers (Supplementary Table S2), and the β-tubulin gene from *A. oligospora* was used as an internal standard. Additionally, the WT strain was incubated on TYGA at 28°C for 2, 3, 5, or 7 days, and hypha was collected for determining the transcription of genes *AovosA* and *AovelB* at different developmental conditions. Similarly, the WT strain was co-cultured with nematodes for 0, 12, 24, or 36 h, and hyphae were collected for determining the transcriptional levels of genes *AovosA* and *AovelB* during the trap formation. The relative transcriptional levels (RTLs) of the candidate genes were calculated as the ratio of the transcript in the mutants over that in the WT strain at a given time using the 2^–Δ^ ^ΔCt^ method (Livak and Schmittgen, 2001).

### Statistical Analyses

One-way analysis of variance (ANOVA) followed by Tukey’s multiple comparison test was used to differentiate the observations, measurements and estimates. *P* < 0.05 were considered to be significantly different. GraphPad Prism version 5.00 (GraphPad Software, San Diego, CA, United States) was used for the photographs and the statistical analyses. Every experiment was repeated three times.

## Results

### Sequence and Phylogenetic Analyses of AoVosA and AoVelB in *A. oligospora*

An online search through the *A*. *oligospora* genome in GenBank using homologous sequences of *A. nidulans* as the queries identified *AovosA* (1494 bp) and *AovelB* (1008 bp). The *AovosA* gene encodes a polypeptide of 497 amino acids (aa) with a predicted isoelectric point (*p*I) and molecular weight (MW) of 9.32 and 54.9 kDa, respectively, whereas the *AovelB* gene encodes a polypeptide of 335 aa with a predicted *p*I and MW of 7.72 and 36.3 kDa, respectively. The deduced AoVosA and AoVelB proteins contain no signal peptides, and both share two conserved domains in their C-termini: the velvet factor (IPR021740) and the velvet domain (IPR037525).

We also downloaded the AoVosA and AoVelB protein homologs from different fungi in GenBank, and compared their amino acid sequence similarity levels using DNAman software (version 6, Lynnon Biosoft, San Ramon, CA, United States). AoVelB shares 94% identity with VelB from *Dactylellina haptotyla*, another NTF, and also shares a high degree of sequence similarity (43.8–61.1%) with orthologous VelB from other filamentous fungi (Supplementary Table S3). Similarly, AoVosA shares 89 and 85.6% identity with homologous VosA from the NTF *D. haptotyla* and *Drechslerella stenobrocha*, respectively, while also sharing mid-range sequence similarity (24–39.1%) with orthologous VosA from other filamentous fungi (Supplementary Table S3).

We constructed a single phylogenetic tree for VosA and VelB from different fungi based on their amino acid sequences, and the orthologous proteins from VosA and VelB in the various fungi clustered as two groups (A and B). The VelB proteins from the different fungi separated into two sub-groups in this tree (A–I and A–II). Specifically, the VelB proteins from two NTF species, *A. oligospora* and *D. haptotyla*, and two species within the *Aspergillus* genus, *A. nidulans*, and *A. fumigatus*, clustered in A–II, whereas the VelB orthologs from other filamentous fungi, including the model fungus *N. crassa*, the phytopathogenic fungus *Magnaporthe oryzae*, the entomopathogenic fungi *Metarhizium anisopliae* and *Beauveria bassiana*, and the nematode-parasitic fungi *Purpureocillium lilacinum*, *Hirsutella minnesotensis*, and *Drechmeria coniospora* clustered in A–I (Supplementary Figure S1). Similarly, the orthologous VosA proteins from the different fungi separated into two sub-groups (B–I and B–II), with three NTF species and two *Aspergillus* genus species clustering in B–I, and orthologs of VosA from other filamentous fungi clustering in B–II (Supplementary Figure S1).

### Screening and Verifying the Δ*AovosA* and Δ*AovelB* Mutants

The pRS426-AoVosA-hph and pRS426-AoVelB-hph plasmids were transformed into the protoplasts of *A. oligospora*, and their transformants were selected on PDAS plates containing 200 μg/mL of hygromycin B (Tunlid et al., 1999). The genomic DNA from each transformant was isolated using the CTAB method and site-specific insertion was confirmed for each plasmid by PCR. Fragments of 2052 and 1804 bp were amplified from the Δ*AovosA* and Δ*AovelB* mutants (Supplementary Figures S2A-b, B-b), respectively, and then compared with the 1356 and 1336 bp fragments from the WT strain using AoVosA-Yf/Yr and AoVelB-Yf/Yr primers (Supplementary Table S1). Finally, three Δ*AovosA* mutants (1, 2, and 6) and three Δ*AovelB* mutants (7, 11, and 22) were obtained. These mutants were further confirmed as authentic by southern blot analysis, and single hybridizing bands were observed in the WT, Δ*AovosA*, and Δ*AovelB* mutants (Supplementary Figures S2A-c, B-c). Their sizes were consistent with our expectations.

### Influence of the *AovosA* and *AovelB* Genes on Growth and Conidiation

**FIGURE 1:**
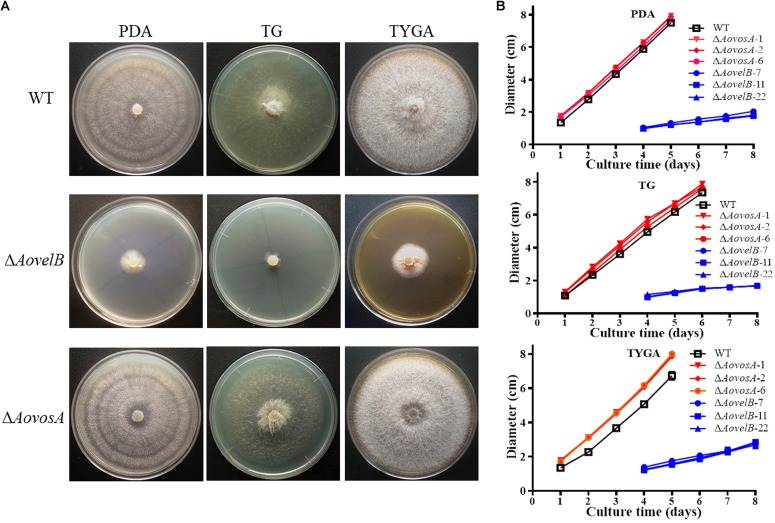
Comparison of colony morphology and mycelial growth between the wild type (WT) and mutant lines. **(A)** Colonies from the WT and mutant lines were incubated on TYGA media for 5 days at 28°C. **(B)** Mycelial growth rates of the WT and mutants on PDA, TYGA, and TG plates.

**FIGURE 2:**
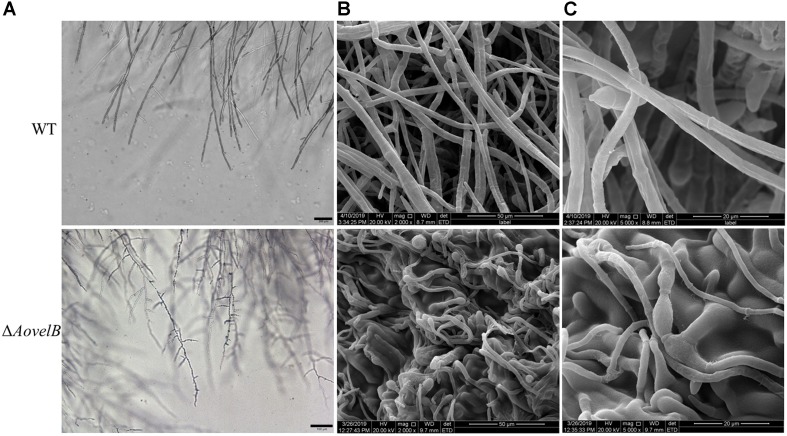
Comparison of mycelial morphology between the wild type (WT) and mutant lines. WT and mutants were cultured for 7 days on CMY plates at 28°C. Mycelial samples were collected and treated as previously described (Kim et al., 2004). **(A)** Mycelial morphologies of the WT and Δ*AovelB* mutants as observed by light microscopy. Bar: 100 μm. **(B,C)** The WT strain and Δ*AovelB* mutant were coated with gold and observed by scanning electron microscopy.

Compared with the WT strain, the Δ*AovelB* mutants showed significant growth defects on PDA, TYGA, and TG media, while the Δ*AovosA* mutants showed no obvious differences in mycelial growth (Figures 1A,B). Colonies produced by the three Δ*AovelB* mutants were denser than those of the WT strain, and their colony diameters were distinctly smaller than those of the WT strain on the three media (Figure 1B). In addition, the aerial hyphae in the Δ*AovelB* colonies were also sparser than those of the WT strain (Figure 1A) and the vegetative hyphae in the Δ*AovelB* colonies were very slender and had many shorter branches than those of the WT strain (Figure 2A). The mycelia were also observed by scanning electron microscopy. While the WT strain had prostrate growth on the surface of the medium, the Δ*AovelB* mutants grew tightly on the medium and the partial cells became expanded (Figures 2B,C).

**FIGURE 3:**
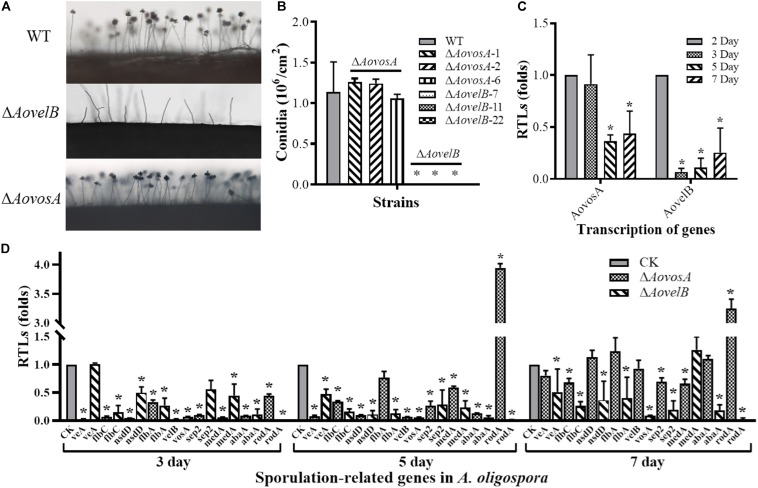
Comparison of conidiation and the transcriptional levels of sporulation-related genes between the wild type (WT) and mutant lines. **(A)** Conidiophore differentiation in the WT and mutant lines. **(B)** Sporulation in the WT and mutant lines on CMY medium. **(C)** The relative transcriptional levels (RTLs) of genes *AovosA* and *AovelB* during the vegetative growth and conidiation in *A. oligospora*. **(D)** RTLs of sporulation-related genes in the mutants compared with the WT strain at different time points. CK. The level of each gene was computed as a ratio of the transcript level of the gene in the deletion mutant to that in the WT strain under a given condition. An asterisk indicates a significant difference between the mutants and the WT strain (*p* < 0.05).

The conidial yields were determined from the WT and mutants after they were cultured in CMY at 28°C for 15 days. Conidial production in the Δ*AovosA* mutants (1.05–1.25 × 10^6^ conidia per cm^2^) were not significantly different compared with the WT strain (1.13 × 10^6^ conidia per cm^2^). The Δ*AovelB* mutants lost the ability to produce conidia, although they did produce conidiophores (Figures 3A,B). The transcription of the *AovosA* and *AovelB* genes was analyzed in the WT strain during the different developmental conditions, including vegetative growth (day 2) and at different time points of conidition, which included the early stage (day 3), middle stage (day 5), and later stage (day 7). The transcriptional levels of *AovosA* and *AovelB* were lower during conidiation than that of vegetative growth. In particular, *AovelB* was significantly down-regulated during the different stages of conidiation (Figure 3C).

The transcriptional levels of 10 sporulation-related genes (*veA*, *flbC*, *nsdD*, *flbA*, *velB*, *vosA*, *sep2*, *medA*, *abaA*, and *rodA*) retrieved from GenBank based on homologous genes in the model fungus *A. nidulans* (Park and Yu, 2012; Krijgsheld et al., 2013), were determined in the WT strain and the Δ*AovelB* and Δ*AovosA* mutants by RT-PCR after culture on PD broth for 3, 5, and 7 days (Figure 3D). The transcriptional levels of all nine genes changed in the Δ*AovelB* mutants compared with those of the WT strain. Of these genes, seven (*flbC*, *nsdD*, *flbA*, *vosA*, *sep2*, *abaA*, and *rodA*) were significantly down-regulated, suggesting a strong interaction between *AovelB* and sporulation-related genes at the transcriptional level. Moreover, the gene transcript of *veA* was down-regulated on days 5 and 7, with no obvious change on day 3. The gene transcript of *medA* was up-regulated on day 7, but was decreased on days 3 and 5 (Figure 3D). Similar to the Δ*AovelB* mutants, the transcriptional levels of the sporulation-related genes, except *rodA*, were down-regulated on days 3 and 5 in the Δ*AovosA* mutant. The transcription of most of the genes was restored to the levels of the WT strain on day 7. The transcription of gene *rodA* was up-regulated 3.94-, and 3.24-fold in the Δ*AovosA* mutants compared with the WT strain on days 5 and 7, respectively (Figure 3D).

### Contributions of AoVosA to Heat Shock Stress

**FIGURE 4:**
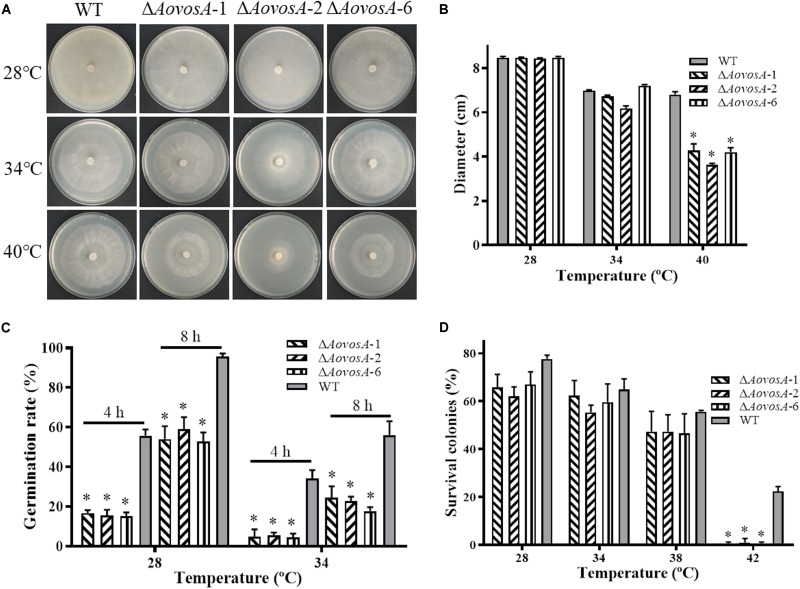
Effect of AoVosA on heat shock stress in *A. oligospora*. **(A)** Colonies from the WT strain and Δ*AovosA* mutants were incubated on PDA for 5 days at 28, 34, and 40°C. **(B)** Mycelial growth rates of the WT and Δ*AovosA* mutants on PDA at the three temperatures. **(C)** Conidia germination rates for the WT strain and Δ*AovosA* mutants at 28 and 34°C. **(D)** Spore survival rate of the WT strain and Δ*AovosA* mutants treated by heat shock at 28, 34, 38, and 42°C. An asterisk indicates a significant difference between the mutants and the WT strain (*p* < 0.05).

The recuperation of the WT strain and Δ*AovosA* mutant was determined, the growth rate of the WT strain and Δ*AovosA* mutants showed no obvious different at 28, 34, or 38°C, while the mycelial growth rate of the Δ*AovosA* mutants was significantly lower than that of the WT strain at 40°C (Figures 4A,B). Further, the spore germination rates of the WT strain and Δ*AovosA* mutants were determined under heat shock stress. The conidia of the WT strain and Δ*AovosA* mutants germinated at 28 or 34°C, but not at 38°C. Conidia from the WT strain germinated significantly faster than those from the cultures of the Δ*AovosA* mutants at 28°C, with approximately 55.7 and 95.7% of the conidia from the WT strain germinating at 4 and 8 h, respectively, while only 15.6 and 55.2% of the conidia from the Δ*AovosA* mutants germinated at the same time points (Figure 4C). Similarly, the spore germination rate of the WT strain was significantly higher than that of the Δ*AovosA* mutants at 34°C, with approximately 34 and 56% of the conidia from the WT strain germinating at 4 and 8 h, respectively, while only 4.77 and 21.5% of the conidia from the Δ*AovosA* mutants germinated (Figure 4C). The surviving spores were determined under different heat shock stresses. The spore survival rate of the WT strain and the Δ*AovosA* mutants showed no obvious differences at 28, 34, or 38°C, while the rates significantly differed at 42°C, with 22.3% survival rate of conidia from the WT strain and essentially none of the spores surviving in the Δ*AovosA* mutants at 42°C (Figure 4D).

### Contributions of AoVosA and AoVelB to Chemical Stress Responses

**FIGURE 5:**
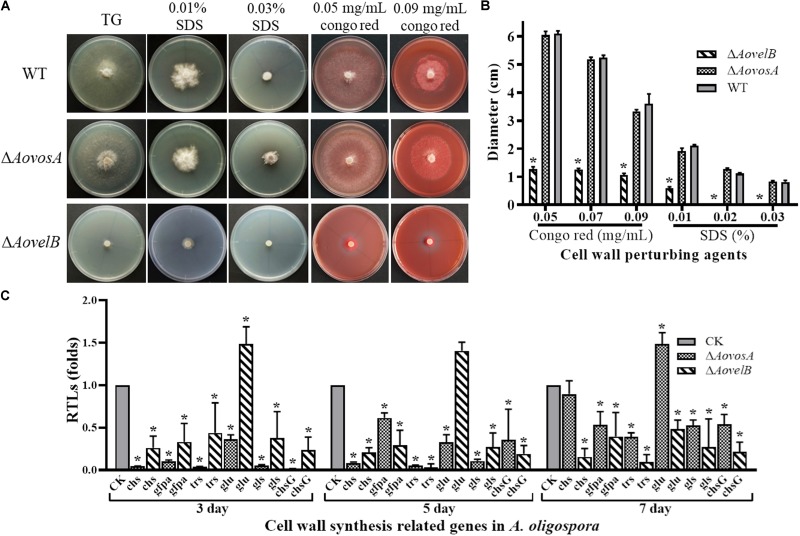
Comparison of stress tolerance to cell wall perturbing agents. **(A)** Colony morphology of the WT and mutants incubated on TG medium supplemented with sodium dodecyl sulfate (SDS) or Congo red. **(B)** The colony diameters of the WT and mutants after incubated on TG medium supplemented with 0.01–0.03% SDS or 0.05–0.09 mg/mL Congo red for 5 days. **(C)** Relative transcriptional levels (RTLs) of cell wall synthesis-related genes in the mutants compared with the WT strain at different time points. CK. The level of each gene was computed as a ratio of the transcript level of the gene in the deletion mutant to that in the WT strain under a given condition. An asterisk indicates a significant difference between the mutants and the WT strain (*p* < 0.05).

The WT strain and the mutants were compared for their responses to six types of chemical agents, including oxidants, cell wall perturbing agents, and osmotic agents. The growth of the WT strain and Δ*AovosA* mutants was significantly inhibited by NaCl and sorbitol. Their colony sizes decreased significantly in response to increasing levels of osmotic agents in the TG plates, unlike the colony sizes of the Δ*AovelB* mutants where no obvious changes were observed on TG medium supplemented with different concentrations of osmotic agents (Supplementary Figure S3). Similarly, the growth of the WT strain and the Δ*AovosA* mutants was inhibited by SDS. The Δ*AovelB* mutants hardly grew on TG plates supplemented with more than 0.02% SDS (Figures 5A,B). In contrast, growth in the WT strain and Δ*AovosA* mutants was inhibited by Congo red, unlike the Δ*AovelB* mutants where no obvious changes occurred when they were grown on TG plates supplemented with 0.05, 0.07, and 0.09 mg/mL Congo red (Figures 5A,B). We next selected six putative genes known to be involved in cell wall synthesis and trehalose synthase (*trs*) (Yang et al., 2018) and measured their transcriptional levels in the WT strain and the Δ*AovosA*, and Δ*AovelB* mutants by RT-PCR. They included the genes encoding chitin synthase (*chs*), glucosamine-fructose-6-phosphate aminotransferase (*gfpa*), β-glucosidase (*glu*), 1,3-β-glucan synthase (*gls*), and chitin synthase G (*chsG*). With the exception of *glu*, which was up-regulated at days 3 and 5, the transcriptional levels of the other genes were down-regulated significantly in the Δ*AovelB* mutants at the tested time points when compared with the WT strain (Figure 5C). Additionally, the transcriptional patterns of these genes in the Δ*AovosA* mutants were similar to the Δ*AovelB* mutants, with most genes down-regulated significantly at the tested time points when compared with the WT strain. However, in the Δ*AovelB* mutant, transcription of *glu* was down-regulated at days 3 and 5, and up-regulated at day 7 (Figure 5C).

**FIGURE 6:**
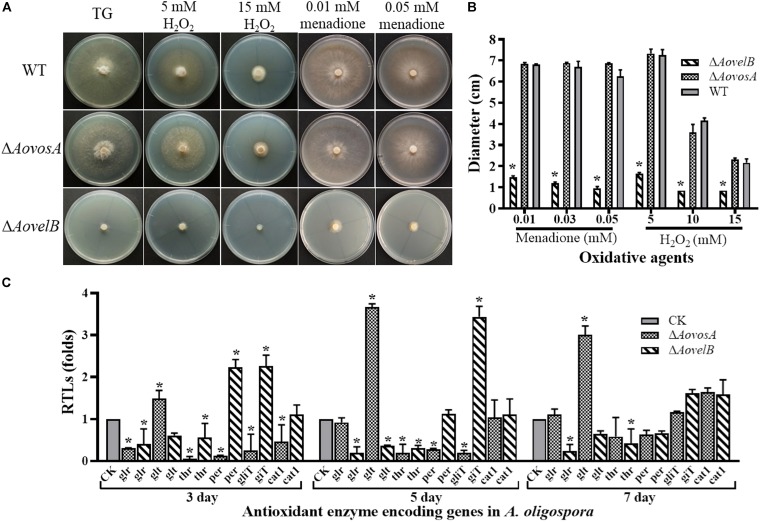
Comparison of stress tolerance to oxidative agents. **(A)** Colony morphologies of the WT and mutant lines incubated on TG medium supplemented with H_2_O_2_ or menadione. **(B)**. Colony diameters of the WT and mutants after incubation on TG medium supplemented with 5–15 mM H_2_O_2_ or 0.01–0.05 mM menadione for 5 days. **(C)** Relative transcriptional levels (RTLs) of oxidation-related genes in the mutants compared with the WT strain at different time points. CK. The level of each gene was computed as a ratio of the transcript level of the gene in the deletion mutant to that in the WT strain under a given condition. An asterisk indicates a significant difference between the mutants and the WT strain (*p* < 0.05).

Growth of the WT strain and the mutants was sensitive to H_2_O_2_. The Δ*AovelB* mutants barely grew on TG agar supplemented with more than 10 mM H_2_O_2_ compared with the WT strain and the Δ*AovosA* mutants (Figures 6A,B). However, the growth of the WT strain and the mutants were not inhibited on TG agar supplemented with 0.01–0.03 mM menadione. Next, we selected six genes encoding the putative proteins involved in antioxidant enzymes for analysis by RT-PCR in the WT strain and the Δ*AovosA* and Δ*AovelB* mutants; these included glutathione reductase (*glr*), glutathione S-transferase (*glt*), thioredoxin reductase (*thr*), peroxidase (*per*), thioredoxin reductase (*gliT*), and catalase (*cat1*). Compared with the WT strain, the transcription of *glr*, *glt*, and *thr* was down-regulated in the Δ*AovelB* mutants. In contrast, transcription of *gliT* was up-regulated, *cat1* was not changed significantly, and the transcription of *per* decreased gradually from days 3 to 7 in the Δ*AovelB* mutants (Figure 6C). Unlike the Δ*AovelB* mutants, the *per* and *gliT* transcripts were down-regulated in the Δ*AovosA* mutants, and the *glt* transcript was significantly up-regulated at all tested time points (Figure 6C).

### AoVelB Plays a Role in Serine Protease Production

**FIGURE 7:**
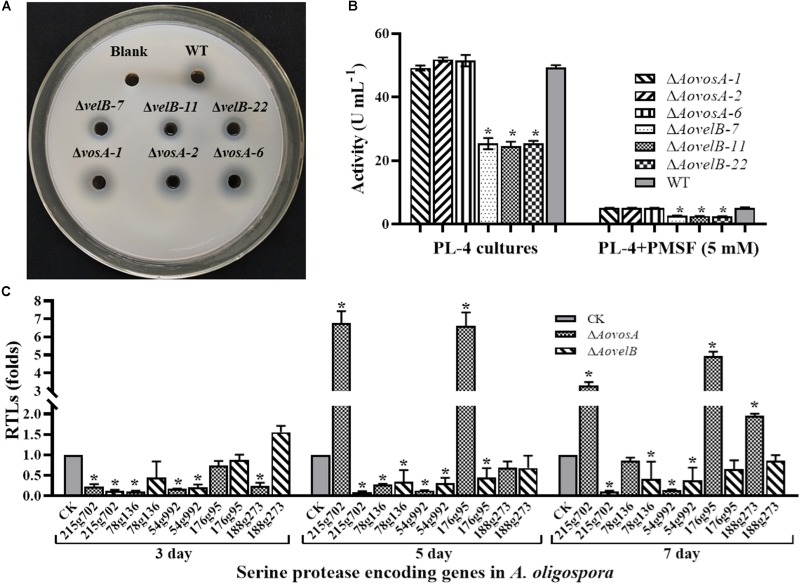
Comparison of extracellular proteolytic activity. **(A)** Comparison of the extracellular proteolytic activities of the WT and mutant lines on casein plates. **(B)** Total extracellular protease activity quantified from 7 days old PL-4 cultures. **(C)** Relative transcriptional levels (RTLs) of serine protease genes in the Δ*AovelB* andΔ*AovosA* mutants compared with the WT strain at different time points. CK. The level of each gene was computed as a ratio of the transcript level of the gene in the deletion mutant to that in the WT strain under a given condition. An asterisk indicates a significant difference between the mutants and the WT strain (*p* < 0.05).

Extracellular serine proteases are important virulence factors in NTF. *A. oligospora*, for example, can produce serine proteases capable of immobilizing nematodes and degrading the proteinaceous components of the nematode cuticle (Tunlid et al., 1994; Yang et al., 2013). Presently, the fermentation broth from the WT strain or from either mutant displayed different proteolytic activities. Compared with the WT strain, disruption of the *AovosA* gene had little influence on the proteolytic activity of the mutant. In contrast the Δ*AovelB* mutants all displayed reduced proteolytic activities (50%) (Figures 7A,B). Additionally, the proteolytic activities of the WT strain and mutants were inhibited by 90% in the presence of the serine protease inhibitor phenylmethyl sulfonyl fluoride (Figure 7C). The transcriptional levels of five of the serine proteases genes in *A. oligospora* were determined by RT-PCR. Of these, *215g702*, *78g136 176g95*, and *54g992* were down-regulated in the Δ*AovelB* mutants. In particular, *215g702* and *54g992* were significantly down-regulated at different time points, while the transcriptional level of *188g273* was slightly up-regulated on day 3 and down-regulated on days 5 and 7 (Figure 7C). Unlike the Δ*AovelB* mutants, the transcriptional levels of all five serine proteases genes were down-regulated on day 3, while the transcription of *215g702* and *176g95* was up-regulated on day 5. An additional gene, *188g273*, was also up-regulated on day 7. The transcription of *54g992* was significant at the three tested times, similar to the Δ*AovelB* mutants (Figure 7C).

### Contributions of AoVosA and AoVelB to Trap Formation in *A. oligospora*

**FIGURE 8:**
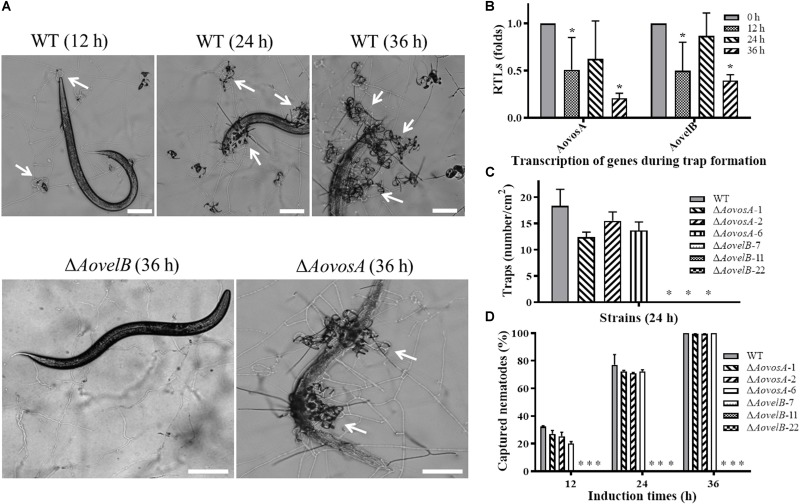
Comparison of trap formation and nematicidal activity in the WT and mutant lines. **(A)** Trap formation in the WT and mutant lines induced by nematodes at different time points. The arrows show the traps produced by the WT strain and Δ*AovosA* mutants. Bar: 100 μm. **(B)** The relative transcriptional levels (RTLs) of genes *AovosA* and *AovelB* in *A. oligospora* during different induction times by nematodes. **(C)** Nematode-induced trap numbers produced by the WT and mutant lines at 24 h. **(D)** The percentage values for the nematodes captured by the WT and mutant lines at different time points. An asterisk indicates a significant difference between the mutants and the WT strain (*p* < 0.05).

Trap formation was induced by adding nematodes to the WA plates. Many traps were observed on the plates with the WT strain and Δ*AovosA*, but no traps were observed on the plates containing the Δ*AovelB* mutants after adding the nematodes for 12, 24, and 36 h (Figures 8A,B). At 12 h, the WT strain and the Δ*AovosA* mutants began to produce immature traps, which only contained one or two circles. Mature traps and three-dimensional nets were formed at 24 and 36 h. Concurrently, approximately 18.3 traps per cm^2^ were observed with the WT strain at 24 h, while 12.3, 15.5, and 13.6 traps per cm^2^ were formed by the three Δ*AovosA* mutants (#1, 2, and 6, respectively) (Figure 8B). The transcription of *AovosA* and *AovelB* was determined during the trap formation. Their expression patterns were similarly down-regulated at the tested time points (Figure 8C). The nematicidal activities of the WT strain and its mutants were also calculated at different time points. Thirty two percent and seventy seven percent of the nematodes were captured by the WT strain at 12 and 24 h, respectively, while 20–27% and 71–72% of the nematodes were captured by the Δ*AovosA* mutants at the same times. By 36 h, almost all of the nematodes were captured, the majority of which were digested by the WT strain and by the Δ*AovosA* mutants. In contrast, the nematodes remained active and were not captured after they were added to the plates containing the Δ*AovelB* mutants for 36 h (Figures 8A,D).

## Discussion

Previous studies have shown that velvet proteins are fungal-specific, multifunctional regulators that control development and secondary metabolism in various filamentous and dimorphic fungi (Bayram et al., 2008; Bayram and Braus, 2012). In the present study, the homologous velvet proteins AoVelB and AoVosA were characterized in *A. oligospora*, a typical NTF species, by gene disruption and by phenotypic and RT-PCR analysis. AoVelB significantly impacted mycelial growth, conidiation, trap formation, and pathogenicity in *A. oligospora*, while AoVosA played limited roles in these biological processes. In addition, we found that the functions of AoVelB and AoVosA in *A. oligospora* also showed several similarities and differences when compared with their homologous proteins in other filamentous fungi.

VelB is required for mycelial growth, but the drastic severity of hyphal morphology defects attributed to the *velB* deletion have not been reported in other filamentous fungi. For example, the slow growth rate and reduced aerial hyphae phenotypes are consistent with observations from the plant pathogen *Fusarium graminearum* (Jiang et al., 2012; Lee et al., 2012). While disruption of the *AovelB* gene resulted in slow growth, reduced aerial hyphae and more branches, concurrently the mycelia grew closely and tightly to the medium, and the partial cells became swollen (Figure 2). In contrast, growth and mycelial morphology in the Δ*AovosA* mutants did not differ from those of the WT strain. These results suggest that AoVelB plays an important role in the vegetative growth of *A. oligospora*.

Apart from defective mycelial growth, AoVelB is evidently essential for sporulation in *A. oligospora* because its mutants totally lost their conidiation ability (Figures 3A,B). The *velB* deletion strains reportedly display decreased conidia production in *A. nidulans* (Park et al., 2012b) and in *Aspergillus flavus* (Chang et al., 2013), but increased conidial yields coupled with immature or low viability conidia in *A. fumigatus* (Park et al., 2012a) and *F. graminearum* (Jiang et al., 2012). However, the conidiation capacity of the Δ*AovosA* mutants did not differ from that of the WT strain (Figures 3A,B). Previous studies have indicated that VosA functions as a negative regulator of conidiation in *A. nidulans* (Ni and Yu, 2007; Sarikaya Bayram et al., 2010) and in *A. fumigatus* (Park et al., 2012a), but is a positive regulator in *F. fujikuroi* (Wiemann et al., 2010). However, this is not the case for AoVosA in *A. oligospora*. Furthermore, *vosA* deletion brought about ascended conidial germination in *A. nidulans* (Park et al., 2012b) and *A. fumigatus* (Park et al., 2012a), but decreased conidial germination in *B. bassiana* (Li et al., 2015). While the conidial morphology of the Δ*AovosA* mutants was unaltered, their conidial germination decreased by 40 and 40.2% at 4 and 8 h at 28°C, respectively, suggesting that AoVosA plays a particular role in conidial germination in *A. oligospora*.

The severe conidiation defects we observed might contribute to the interactions that occur between AoVelB and other conidiation regulators. For example, deleting the *AovelB* gene led to the transcriptional depression of almost all of the conidiation regulators. From these regulators, seven genes (*flbC*, *flbA*, *nsdD*, *abaA*, *vosA*, and *rodA*) were significantly down-regulated (Figure 3D). These genes are crucial for *A. nidulans* conidiation (Park and Yu, 2012). In particular, AbaA is considered to be a central developmental regulator that can activate the expression of *vosA* and *velB* in *Aspergillus* phialides (Park and Yu, 2012). Interestingly, despite having little influence on conidiation, the transcription levels of these sporulation-related genes, except for *rodA*, were also significantly down-regulated on days 3 and 5 in the Δ*AovosA* mutants. The transcription of most genes was restored to the level of the WT strain on day 7. The transcription of *rodA* was significantly different between the Δ*AovelB* and Δ*AovosA* mutants on days 5 and 7, being down-regulated in the Δ*AovelB* mutants and up-regulated in the Δ*AovosA* mutants (Figure 3D). The *rodA* gene encodes a hydrophobin and is essential for formation of the rodlet layer and hydrophobicity in conidia. Conidia of the *rodA* null mutants are hydrophilic due to the absence of the outermost rodlet layer (Thau et al., 1994).

Apart from the abolished conidiation capacity, the sensitivity of the Δ*AovelB* mutants to oxidative and cell wall perturbation stresses increased under H_2_O_2_ and SDS stresses, suggesting that AoVelB is also required to resist such stresses. Previous research findings in *A. fumigatus* (Park et al., 2012a) are largely consistent with our observations in *A. oligospora* but are contrary to those in *F. graminearum*, whose resistance to osmotic stress and cell wall-damaging agents is greater (Jiang et al., 2012). In contrast, the Δ*AovosA* mutants showed no change in their resistance to chemical agents compared with the WT strain (Figures 5, 6 and Supplementary Figure S3). These phenotypic changes in the Δ*AovosA* mutants do not coincide with those reported in *A. nidulans*, whose Δ*vosA* mutants were highly sensitive to the H_2_O_2_ oxidant (Ni and Yu, 2007). Therefore, the AoVosA and AoVelB protein homologs from different fungi might play diverse functions, and their roles likely vary in fungal species.

The spore germination rate of the Δ*AovosA* mutants was significantly lower than that of the WT strain at 28 or 34°C, the proportion of germinated spores in the Δ*AovosA* mutants was 28% compared with the WT strain at 28°C (4 h), but the proportion was decreased to 14% at 34°C for 4 h (Figure 4C). Additionally, the recuperation of the Δ*AovosA* mutants was significantly lower than that of the WT strain at 40°C (Figures 4A,B). Moreover, the spore survival rate of the WT strain was significantly higher than that of the Δ*AovosA* mutants at 42°C (Figure 4D). These results suggest that the AoVosA protein plays a role in conidial germination and heat shock stress resistance in *A. oligospora*. Our results coincide with those reported in *A. nidulans* (Ni and Yu, 2007) and *B. bassiana* (Li et al., 2015). For example, the deletion of *vosA* in *A. nidulans* results in the lack of trehalose in spores; and rapid loss of the cytoplasm, organelles, and viability of spores; and a dramatic reduction in tolerance of conidia to heat (Ni and Yu, 2007).

Presently, *A. oligospora* began to produce spores on day 4 during incubation on TYGA at 28°C. Spores were abundant by day 6 and peaked in number on day 8. Given that gene expression occurred prior to the corresponding phenotype, the mycelia collected on day 2 were specified as the vegetative growth stage, while mycelia collected on days 3, 5, and 7 were defined as the early stage, middle stage, and later stage of conidiation, respectively. The transcription of *AovosA* and *AovelB* during the different developmental conditions was determined by RT-PCR. Both genes were highly expressed in the vegetative growth stage, but their transcription was down-regulated at the early stage of conidiation and increased at the later stage of conidiation (Figure 3C). These results are consistent with the prior finding in *A. nidulans*, in which the transcript levels of genes *vosA* and *velB* were high in conidia and detectable during vegetative growth and increased from 48 h post-developmental light-mediated induction (Eom et al., 2018).

Furthermore, the transcriptional levels of several genes involved in cell wall biosynthesis were down-regulated in the Δ*AovelB* and Δ*AovosA* mutants (Figure 5C). Of these, decreased levels of the *trs* transcript were observed in both the Δ*AovelB* and Δ*AovosA* mutants. The gene *trs* contributes to trehalose synthesis, which is critical for conidia to resist multiple stresses, such as SDS treatment (Ni and Yu, 2007; Zhang and Feng, 2018). Additionally, the transcription pattern of *glu* was opposite in the Δ*AovelB* and Δ*AovosA* mutants, suggesting that *glu* might be a key gene for cell wall biosynthesis. Analogously, the transcripts from three genes related to antioxidant processes were down-regulated in the Δ*AovelB* mutants. Previous studies have confirmed that sulfhydryl groups play essential roles in oxidative stress responses (Grant, 2001). Moreover, the transcripts of genes related to antioxidant, with the exception of *glt*, in the Δ*AovosA* mutants were down-regulated on days 3 and 5, and no obvious difference was observed between the WT strain and mutants on day 7. In contrast, the transcription of *glt* was significantly up-regulated at all tested time points (Figure 6C). Glutathione S-transferase encoded by *glt* is involved in the oxidative stress response in *Schizosaccharomyces pombe* (Kim et al., 2001).

Pathogenic fungi often secrete a series of virulence factors (e.g., mycotoxins and serine proteases) into the extracellular environment to facilitate infection and allow the pathogen to assimilate essential nutrients from the host (Yang et al., 2013). The extracellular proteolytic activities of the Δ*AovelB* mutants were reduced when compared with the WT strain, and the transcripts of three serine protease genes (*215g702*, *78g136*, and *54g992*) were clearly down-regulated, suggesting that AoVelB is involved in regulating serine protease production. The protein encoded by the *215g702* gene is a cuticle-degrading serine protease capable of degrading the proteinaceous components of nematodes (Yang et al., 2011). The transcripts of serine protease genes were also down-regulated in the Δ*AovelB* mutants on day 3, while partial genes were up-regulated on days 5 and 7. Especially, the transcripts of *215g702* and *176g95* were significantly up-regulated. Additionally, the proteolytic activities of the WT strain, Δ*AovosA* and Δ*AovelB* mutants were significantly inhibited by phenylmethyl sulfonyl fluoride (Figure 7B), suggesting that the main extracellular proteases are serine proteases (Tunlid et al., 1994; Yang et al., 2013).

Notably, deletion of the *AovelB* gene abolished the ability of *A. oligospora* to produce the traps required to capture nematodes, suggesting that AoVelB plays a vital role in the biological control potential of *A. oligospora*. While reduced numbers of traps were produced by the Δ*AovosA* mutants at 24 h, their nematicidal activities were lower than those of the WT strain at 12 and 24 h, but identical to the WT strain at 36 h (Figures 8C,D). Additionally, the transcripts of *AovelB* and *AovosA* showed a similar pattern, in which both were down-regulated at the early stage of trap formation (12 h), increased at the middle stage (24 h), but decreased at the later stage of trap formation (36 h) (Figure 8B). Previous studies revealed that VosA and VelB play important roles in either conidiation or secondary metabolism, which may also affect the pathogenicity of filamentous fungi, such as the entomopathogen *B. bassiana* whose conidia can infect insects (Li et al., 2015), while *F. graminearum* (Jiang et al., 2012) and *F. fujikuroi* (Wiemann et al., 2010) produce mycotoxins that can poison grains. *A. oligospora* is a typical NTF species, and trap formation by it is considered an important indicator of the *A. oligospora* lifestyle transition (Su et al., 2017). These observations suggest that trap formation defects result from the absence of AoVelB, which probably causes serious hyphal defects, making it impossible to produce the traps formed by mycelium development and differentiation.

Previous studies have confirmed that VosA and VelB can form a VosA–VelB heterodimer, which plays important roles in spore viability, conidial maturation, and germination (Ni and Yu, 2007; Ahmed et al., 2013; Park et al., 2017). In this study, the transcription of *vosA* was significantly down-regulated in the Δ*AovelB* mutants and, in contrast, the transcription of *velB* was significantly down-regulated in the Δ*AovosA* mutants. These findings suggest that VosA interacts with VelB at the transcriptional level. At present, no suitable markers are available to construct a double mutant, which hinders studies of the biological function of key genes in *A. oligospora*, such as *AovosA* and *AovelB*. Identification of a novel selectable marker is an important goal.

Our collective results demonstrate that AoVelB is a crucial regulator of multiple biological processes, including mycelial growth, conidiation, trap formation, and serine protease production in *A. oligospora*, whereas AoVosA plays a role in conidial germination and heat shock stress, with a minor role in trap formation. The data provide the first characterization of AoVelB and AoVosB in a typical NTF species, *A. oligospora*. Our results provide a basis for further exploration of the mechanism whereby AoVelB regulates conidiation, trap formation, and other phenotypic traits, and will inform investigations of the functions of velvet proteins in NTF to provide a fuller picture of their biological roles.

## Conclusion

We identified and characterized the AoVosA and AoVelB velvet proteins from *A. oligospora*. AoVelB plays a role in conidiation and is important for trap formation, as well as in infection-related morphogenesis in this fungus. AoVosA plays a role in conidial germination and heat shock stress. Our findings enhance current understanding of conidiation, trap formation and the pathogenic mechanisms of NTF.

## Data Availability

The datasets generated for this study can be found in GenBank, AOL_s00054g700 and AOL_s00054g811.

## Author Contributions

JY and K-QZ conceived and designed the study. GZ, YZ, and JY wrote the manuscript and conducted the experiments. YM, LY, MX, and DZ analyzed the data. JY and XN revised the manuscript. All authors read and approved the final manuscript.

## Conflict of Interest Statement

The authors declare that the research was conducted in the absence of any commercial or financial relationships that could be construed as a potential conflict of interest.

## Footnotes

1 https://www.ncbi.nlm.nih.gov/genbank/
