# Supplementary material for: The Velvet Proteins VosA and VelB Play Different Roles in Conidiation, Trap Formation, and Pathogenicity in the Nematode-Trapping Fungus Arthrobotrys oligospora
Source: Front Microbiol. 2019 Aug 20;10:1917. doi: 10.3389/fmicb.2019.01917 (PMC6710351; doi:10.3389/fmicb.2019.01917)
Supplement: Supplementary file 1 [file Data_Sheet_1.docx]

**Supplementary materials**

**1. Supplementary Figures**

**Supplementary Figure S1** **Phylogenetic analysis based on the deduced amino acid sequences of homologous VelB and VosA from different fungi.** GenBank accession numbers are given in brackets. These proteins were divided into two groups (A and B).


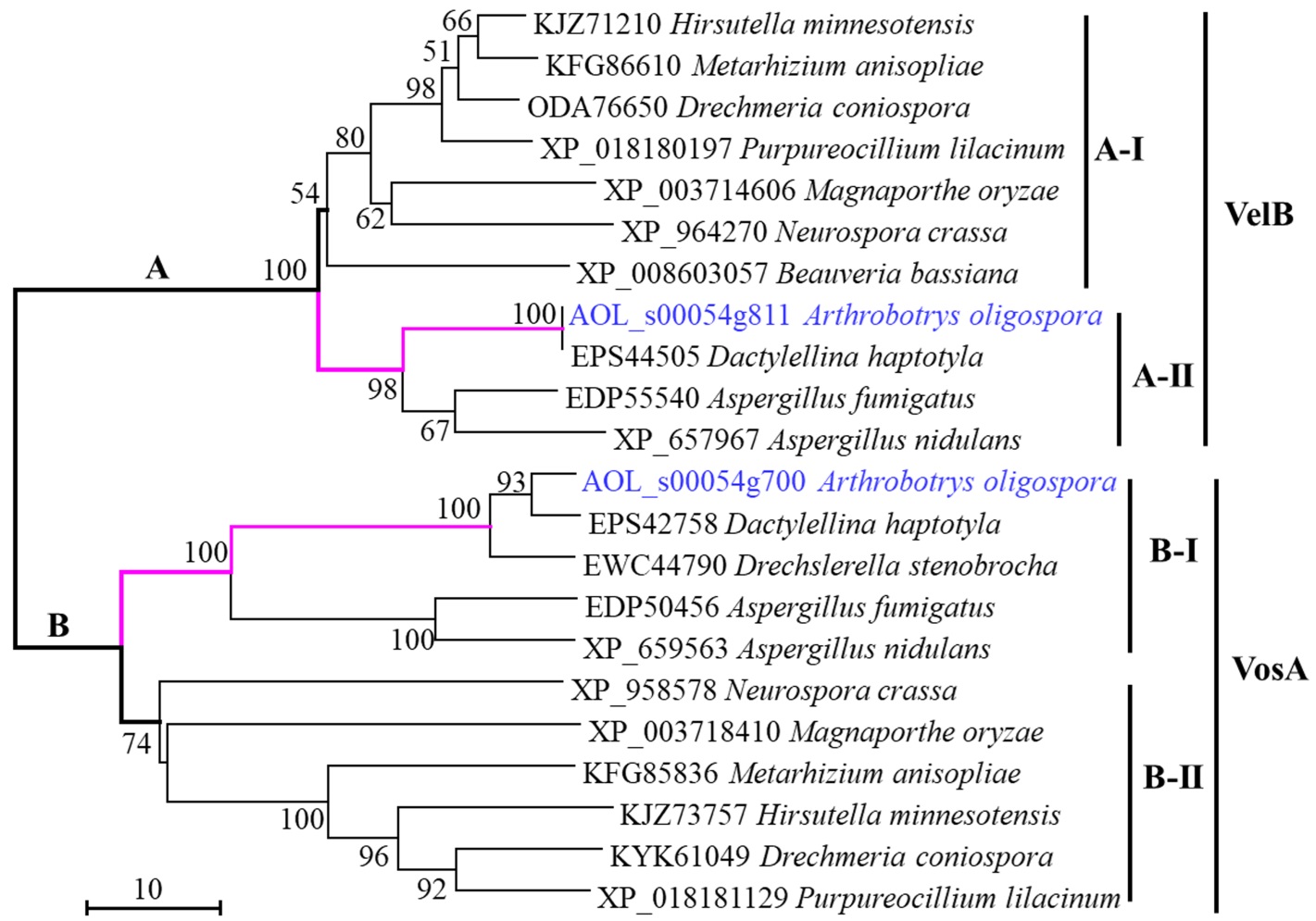


**Supplementary Figure S2 Knock-out and verification of genes *AovelB* and *AovosA* in *A. oligospora*.** A. Verification of the knock-out of gene *AovosA* using PCR method and Southern blot. A-a, Diagram of homologous recombination of gene *AovosA*, the homologous flanks of the target gene, southern blotting probe, and the sites of restriction enzyme *Kpn*I are marked. A-b, the gene *AovosA* mutants were confirmed by PCR. The numbers 1, 2, and 6 denote the positive transformants, and WT denotes the wild-type (WT) strain. M denotes the DNA marker. A-c, the gene *AovosA* mutants were confirmed by southern blotting analysis. B. Verification of the knock-out of gene *AovelB* using PCR and southern blot. B-a, Diagram of homologous recombination of gene *AovelB*, the homologous flanks of the target gene, southern blotting probe, and the sites of restriction enzyme *Xba*I are marked. B-b, the gene *AovelB* mutants were confirmed by PCR. The numbers 7, 11, and 22 denote the positive transformants. B-c, *AovelB* mutants were confirmed by southern blotting.


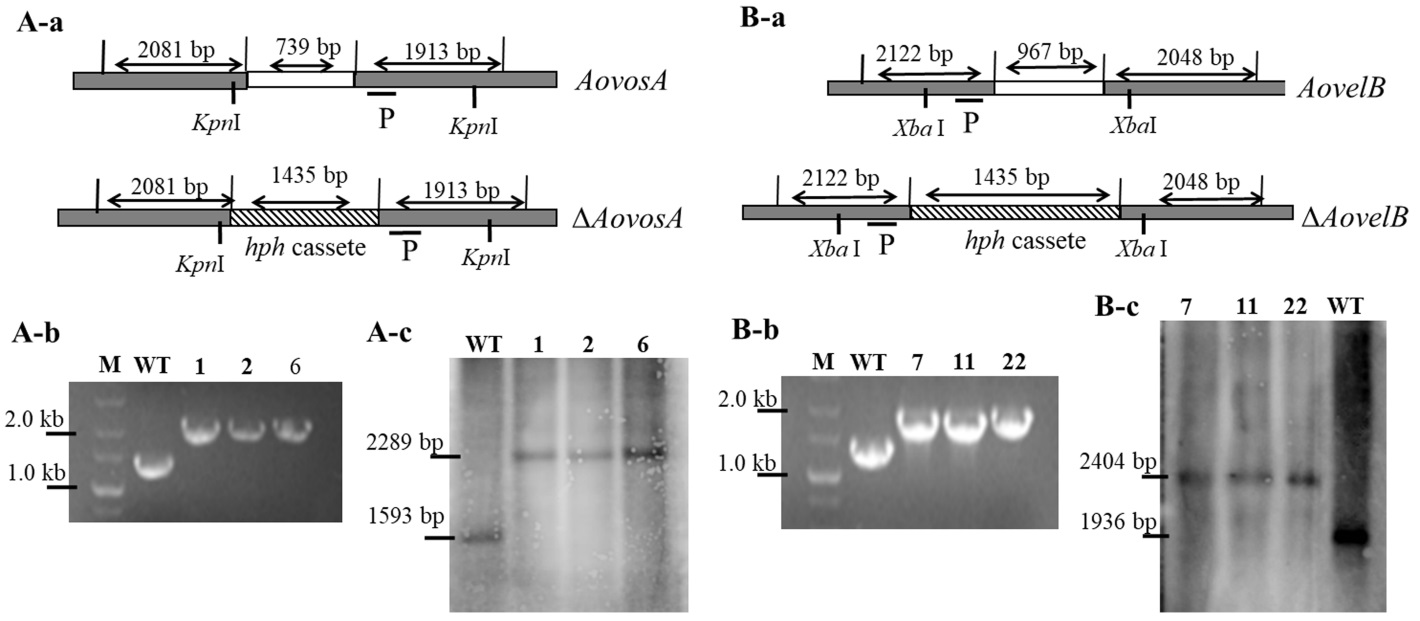


**Supplementary Figure S3 Comparison of stress tolerance to osmotic agents.** A. Colony morphologies of the wild type (WT) strain and mutants incubated on TG medium supplemented with NaCl or sorbitol. B. Colony diameters of the WT and mutants after incubation on TG medium supplemented with 0.10-0.30 M NaCl or 0.25-0.75 M sorbitol for 7 days.


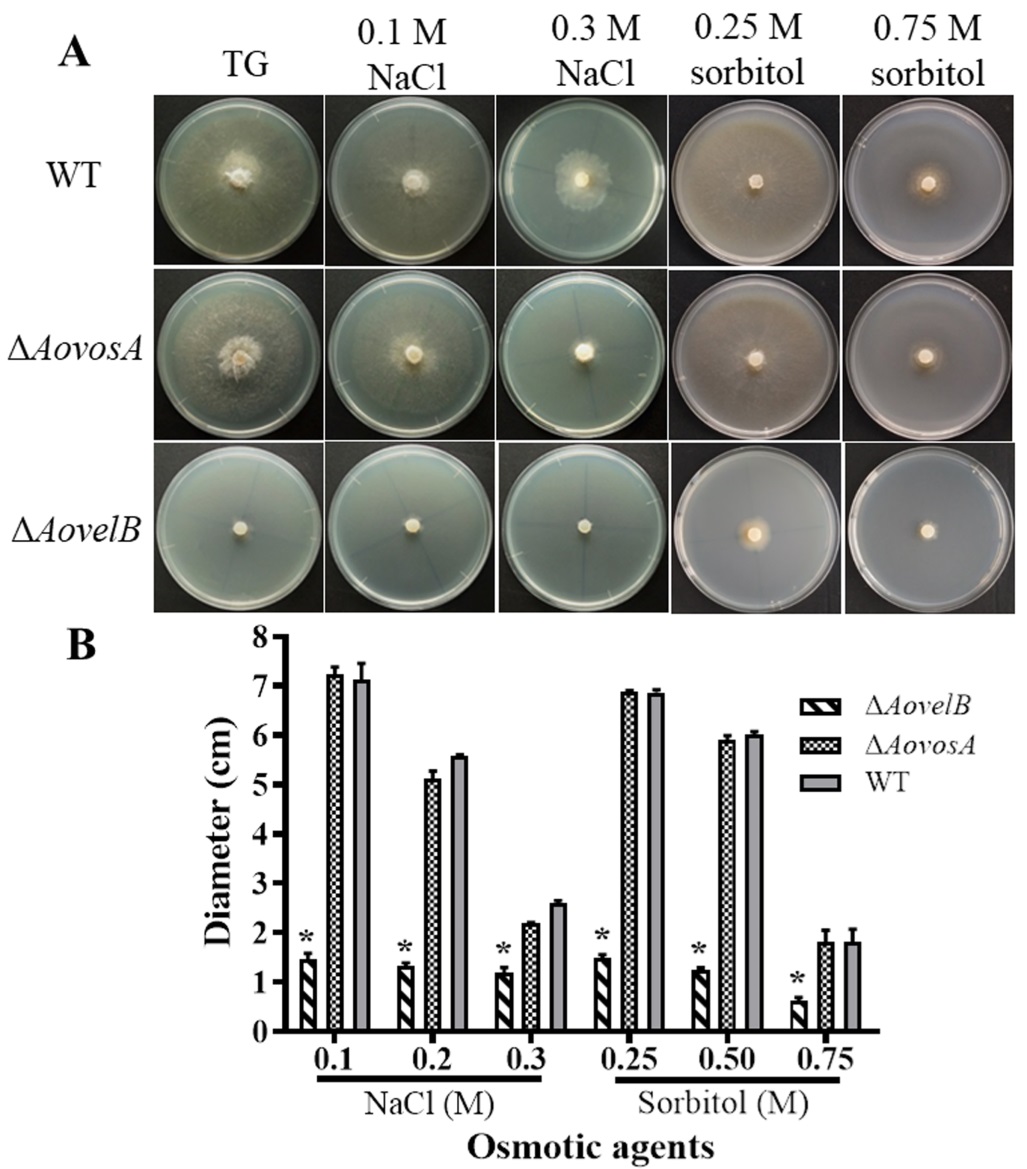


**2. Supplementary Tables**

**Supplementary Table S1. List of primers used in this study.**

| Primers | Sequence (5′-3′) | Description |
| --- | --- | --- |
| *vosA*-5f | GTAACGCCAGGGTTTTCCCAGTCACGACGACCAGTCGACGTCCTACCAA | Amplify the *AovosA* gene 5′ flank |
| *vosA*-5r | ATCCACTTAACGTTACTGAAATCTCCAACGGCTAAGGAGGAACAGTTGC |  |
| *vosA*-3f | CTCCTTCAATATCATCTTCTGTCTCCGACCCCGGAATGTCAGAGTCTAC | Amplify the *AovosA* gene 3′ flank |
| *vosA*-3r | GCGGATAACAATTTCACACAGGAAACAGCTTGCATCAGTGTGGTGTGTG |  |
| *velB*-5f | GTAACGCCAGGGTTTTCCCAGTCACGACGACCAGACCAAAACCTCACC | Amplify the *AovelB* gene 5′ flank |
| *velB*-5r | ATCCACTTAACGTTACTGAAATCTCCAACTAGATGCTTCGCGACTCAG |  |
| *velB*-3f | CTCCTTCAATATCATCTTCTGTCTCCGACTTTGTCTCCAAACCCACACA | Amplify the *AovelB* gene 3′ flank |
| *velB*-3r | GCGGATAACAATTTCACACAGGAAACAGCCCTGCAAGGAGGAGATCTTG |  |
| hphF | GTTGGAGATTTCAGTAACGTTAAGTGGAT | Amplify the *hph* cassette |
| hphR | GTCGGAGACAGAAGATGATATTGAAGGAGC |  |
| *vosA*-Yf | CTGGTTTCGCTGCGTTCG | Amplify the positive transformant |
| *vosA*-Yr | TTTGATTTGGTGGTGGGTG |  |
| *velB*-Yf | GCCATAGAGCGCAATAGAGT |  |
| *velB*-Yr | CATCCATTTCATCCATACGC |  |
| *vosA*-T5 | CAGAAAGGAACCCCGAACTC | Make Southern blotting probe |
| *vosA*-T3 | TGAGGGTGTTGCGAATGTG |  |
| *velB*-T5 | CGACTGTGACCGCCAGTTTA |  |
| *velB*-T3 | GGACGATGATGACGATTACTGA |  |

**Supplementary Table S2. Paired primers used for RT-PCR of phenotype related genes, such as conidiation, stress tolerance and serine proteases, in *A. oligospora*.**

| Cell wall synthesis genes | Sequence  (5′-3′) | Serine protease  genes | Sequence  (5′-3′) |
| --- | --- | --- | --- |
| AOL_s00078g76  (*chs*) | 76-5F-GCCACTCTGCCATCTTTAGC  76-3R-GCATCTTCACCCGCACCAGT | AOL_s00078g136  (*78g136*) | 136-5F-ACACTTGCCCATTTCACTCC  136-3R-GCTGGGTTTCACAACATCCT |
| AOL_s00076g99 (*gfpa*) | 99-5F-CCATCATCGAGCACACCAAG  99-3R-CCACCGAGAGTCACTGTCTT | AOL_s00188g273  (*188g273*) | 273-5F-GTGGATGAAACCCGGATATG  273-3R-CTCCCACTATCACCGCAACT |
| AOL_s00097g268  (*trs*) | 268-5F-CACGTCCATATCACCCTCGA  268-3R-GTGGTATCGGCGACAGTTTC | AOL_s00215g702  (*215g702*) | 702-5F-GTCGCCGCTGACTTAACTGT  702-3R-ATAATTGCTGATTCGCTGGG |
| AOL_s00083g375  (*glu*) | 375-5F-GTCATCCTCAAGAACGTCGC  375-3R-ATATGGAAAGTTGGCCGTGC | AOL_s00054g992  (*54g992*) | 992-5F-TCCGCAACTTCAAGAGTGTG  992-3R-CGTTGGCTTCCTCGTTAGAG |
| AOL_s00054g491  (*gls*) | 491-5F-AGCTCTGTTCTGGTGATGCT  491-3R-GATGTTTCGCCAAGGACTCC | AOL_s00176g95  (*176g95*) | 95-5F-CCCAAAAAGAATGCACCACT  95-3R-AGAGGCGGGAAAATACCTGT |
| AOL_s00075g119  (*chsG*) | 119-5F-TCCCTCCAGTCCAGATGATG  119-3R-TCTTGAGACCTCGACCCAAC |  |  |
| Sporulation genes | Sequence (5′-3′) | Oxidants-degrading genes | Sequence (5′-3′) |
| AOL_s00169g18  (*veA*) | 18-5F-AAGCTACACCCAATCAACGC  18-3R-TTGCGATGCTGACGATCTTG | AOL_s00054g13  (*glr*) | 13-5F-CGAAAAGTCTGAATCGGGTGA  13-3R-GCTCCACTTTGCCACATACATC |
| AOL_s00075g211  (*nsdD*) | 211-5F-ATTACGGCCGCCTAGTAGTC  211-3R-CTCGTTTGGACCTGGTTGTG | AOL_s00169g61  (*glt*) | 61-5F-AGAAACCTACGACCCAAACCA  61-3R-CCCTTCCACCTCCTGAACTT |
| AOL_s00215g516  (*flbA*) | 516-5F-TTCAAACGCAGCTCCTTCAC  516-3R-AAGCGGGTTGACAGATGAGA | AOL_s00078g209 (*thr*) | 209-5F-TGTCATCGGTGGTGGAGATT  209-3R-ACAAGAGAGGTAGCGGGTTC |
| AOL_s00006g570  (*rodA*) | 570-5F-GCGGATCCAACATGAAGCTT  570-3R-GGTTGACAACTGGGATGCTG | AOL_s00215g326  (*per*) | 326-5F-CACCATCCGCTCTGTCTTCA  209-5F-TGTCATCGGTGGTGGAGATT |
| AOL_s00054g700  (*vosA*) | 700-5F-CAAACCACCCACCACCAAAT  700-3R-GGATGGACAGGAGAAGGACC | AOL_s00054g257  (*gliT*) | 257-5F-TTTCGGTCAAGAGCAAAAGTG  257-3R- GCTATCATTCCATTCCCCATT |
| AOL_s00007g157 (*flbC*) | 157-5F-CTCTCCGGCAAAGACAATCG  157-3R-GTCGACTGAGGATAGTAGCT | AOL_s00173g374  (*cat1*) | 374-5F- TCCCCATCCTCATCCATACG  374-3R- GATAGCGGGCATTTCTTTCC |
| AOL_s00210g120  (*medA*) | 120-5F-TCCGGCCCAATGATTCAGAA  120-3R-AGATCGCAGGAACATGGTGA |  |  |
| AOL_s00080g63 (*abaA*) | 63-5F-AACTTTATGCGCCTTGTCGT  63-3R-TTGGCTAGGTGGTCTGTACG |  |  |
| AOL_s00054g811  (*velB*) | 811-5F- ATTCCGCAACTTCTCCCTCA  811-3R- GGCATGTTTGGATTCTGGGG |  |  |
| AOL_s00215g893 (*sep2*) | 893-5F-ATACCGCCAACACCCTCTAC  893-3R-AACCATCTTCATCTCGGCCT |  |  |

| β-tubulin gene | Sequence (5′-3′) |  |  |
| --- | --- | --- | --- |
| AOL_s00076g640 (*tub*) | tubA-F-CCACCTTCGTCGGTAACTC  tubA-R-TCGTCCATACCCTCACCAG |  |  |

**Supplementary Table S3. Homologous analysis of AoVelB and AoVosA with homologs from other filamentous fungi.** The similarity of homologous AoVelB and AoVosA from different fungi was analyzed using the DNAman software package (Version 5.2.2, Lynnon Biosoft, Canada).

| **Fungi** | **GenBank nos.** | **Identities (%)** |
| --- | --- | --- |
| ***Arthrobotrys oligospora*** | **AOL_s00054g811 (AoVelB)** |  |
| *Dactylellina haptotyla* | EPS44505 | 94.0 |
| *Aspergillus fumigatus* | EDP55540 | 61.1 |
| *Aspergillus nidulans* | XP_657967 | 55.6 |
| *Magnaporthe oryzae* | XP_003714606 | 51.4 |
| *Purpureocillium lilacinum* | XP_018180197 | 50.3 |
| *Drechmeria coniospora* | ODA76650 | 55.1 |
| *Neurospora crassa* | XP_964270 | 50.0 |
| *Beauveria bassiana* | XP_008603057 | 44.0 |
| *Hirsutella minnesotensis* | KJZ71210 | 44.6 |
| *Metarhizium anisopliae* | KFG86610 | 43.8 |
| **Fungi** | **GenBank nos.** | **Identities (%)** |
| ***Arthrobotrys oligospora*** | **AOL_s00054g700 (AoVosA)** |  |
| *Dactylellina_haptotyla* | EPS42758 | 89.0 |
| *Drechslerella_stenobrocha* | EWC44790 | 85.6 |
| *Aspergillus fumigatus* | EDP50456 | 39.1 |
| *Magnaporthe_oryzae* | XP_003718410 | 27.8 |
| *Aspergillus_nidulans* | XP_659563 | 36.2 |
| *Hirsutella_minnesotensis* | KJZ73757 | 33.8 |
| *Drechmeria_coniospora* | KYK61049 | 35.3 |
| *Purpureocillium_lilacinum* | XP_018181129 | 35.2 |
| *Neurospora crassa* | XP_958578 | 24.0 |
| *Metarhizium anisopliae* | KFG85836 | 32.7 |
